# Benign Solitary Pulmonary Necrotic Nodules: How Effectively Does Pathological Examination Explain the Cause?

**DOI:** 10.1155/2020/7850750

**Published:** 2020-07-04

**Authors:** Halide Nur Urer, Mehmet Zeki Gunluoglu, Nurcan Unver, Sezer Toprak, Mediha Gonenc Ortakoylu

**Affiliations:** ^1^University of Health Sciences Turkey, Yedikule Chest Diseases and Thoracic Surgery Training and Research Hospital, Department of Pathology, Istanbul, Turkey; ^2^Medipol University School of Medicine, Thoracic Surgery, Istanbul, Turkey; ^3^University of Health Sciences Turkey, Yedikule Chest Diseases and Thoracic Surgery Training and Research Hospital, Department of Microbiology, Istanbul, Turkey; ^4^University of Health Sciences Turkey, Yedikule Chest Diseases and Thoracic Surgery Training and Research Hospital, Department of Pulmonary Medicine, Istanbul, Turkey

## Abstract

**Aims:**

We investigated the histopathological features of solitary pulmonary necrotic nodules (NNs) of undetermined cause. We combined our findings with those obtained using other methods to determine how well the etiological factors were explained.

**Methods:**

We screened patients who underwent surgery to treat solitary pulmonary granulomatous and nongranulomatous NNs of undetermined cause. The NN sizes and features of both the NNs and adjacent parenchyma were evaluated. Histochemical analyses included Ehrlich–Ziehl–Neelsen (EZN), Grocott, and Gram staining. Polymerase chain reaction (PCR) was used to detect tuberculous and nontuberculous mycobacteria, panfungal DNA, *Nocardia*, *Francisella tularensis* types A and B, and actinomycetes.

**Results:**

The NNs were granulomatous in 78.9% and nongranulomatous in 21% of the 114 patients included. EZN staining or PCR was positive for *Mycobacterium* in 53.5% of all NNs: 62.2% of granulomatous and 20.8% of nongranulomatous NNs. We found a weak but significant correlation between granulomatous NNs and *Bacillus* positivity and a significant correlation between granulomas surrounding the NNs and the presence of multiple necroses. The NN etiology was determined via histopathological, histochemical, and PCR analyses in 57% of patients but remained undetermined in 42.9%.

**Conclusion:**

The causes of both granulomatous and nongranulomatous NNs can be determined by pathological examination. Granulomatous necrosis and granulomas in the adjacent parenchyma are important for differential diagnosis. When both features are present, they strongly support a diagnosis of tuberculosis, even in the absence of bacilli.

## 1. Introduction

Pulmonary necrotic nodules (NNs) are space-occupying lung lesions. The principal etiologies include infection, collagen tissue disease, thromboembolism, vasculitis, and aspiration. NNs are often difficult to distinguish from malignancies on clinical examination and radiological imaging. The underlying cause of pulmonary NNs can sometimes be determined based on morphology. Identification of an infectious agent facilitates diagnosis. However, the causes of some benign pulmonary nodules cannot be determined.

Here, we explored how often the etiological factors can be identified. The diagnostic and histopathological characteristics of the lesions were defined and evaluated in combination with histochemical and polymerase chain reaction (PCR) analyses.

## 2. Materials and Methods

Cases of benign pulmonary NNs and granulomatous NNs treated between January 1, 2015, and December 31, 2017, in the Pathology Department of Yedikule Pulmonary Diseases and Thoracic Surgery Training and Research Hospital were screened. Archived slides and pathology reports were analyzed. Patients who received specific diagnoses based on microbiological and serological tests performed during clinical evaluation were excluded. Patients in whom the etiology was identified by routine histopathological evaluation, those with synchronous tumors, and those who received neoadjuvant therapy were also excluded. Age, sex, and type of surgical resection were recorded in all cases. A total of four sections of 3 *μ*m thickness were obtained from one or two paraffin blocks. We performed routine hematoxylin-and-eosin, Ehrlich–Ziehl–Neelsen (EZN), Gram, and Grocott staining. All slides were examined under a light microscope by two pathologists (H.N.U. and N.U.). Both our hospital institutional review board and our local ethics committee approved the study.

### 2.1. Histopathological Evaluation

The NNs were divided into two groups: granulomatous NNs (those exhibiting epithelioid histiocytes in the periphery) and nongranulomatous NNs (all others). The nature and size of each NN and the surrounding tissue features were evaluated. Necrosis within an NN was recorded in terms of size (≤1 or >1 cm), border type (smooth or irregular), shape (round, triangular, or amorphous), necrotic quality (caseified, infarctoid, apoptotic, or “dirty” with many polymorphonuclear leukocytes), number (single or multiple), and location. The parenchyma surrounding each NN was evaluated in terms of granuloma status, vasculitis (granulomatous, lymphoplasmacytic, necrosis, and leukocytoclastic), parenchymal changes, and foreign body and thromboembolism statuses. NNs positive on PCR and/or EZN staining were considered to contain *Mycobacterium tuberculosis* (MT).

### 2.2. Histochemical Evaluation

Each slide was stained with EZN, Grocott, and Gram stains. The entire sample area was observed under a light microscope at 40× magnification. When required, 100× magnification was used for closer examination. Acid-fast bacilli (AFB) were assessed by EZN staining, fungal spores and hyphae by Grocott staining, and Gram-positive and -negative bacteria by the Gram staining.

### 2.3. PCR Procedures

Necrotic areas were marked in one or two paraffin blocks. Microdissection was performed under sterile conditions. The samples were transferred to coded, sterile Eppendorf tubes. After deparaffinization, DNA was isolated using the InstaGene Matrix kit (Bio-Rad, USA). MT and nontuberculous mycobacteria were detected using a commercial kit (GenMark, Turkey). Each tube contained 10 *μ*L Real-time PCR Master Mix with UDG, 5 *μ*L Primer Probe Mix, and purified DNA (5 *μ*L). PCR was performed using the CFX96 Real-time PCR device (Bio-Rad). The following primer sets specific to panfungal, *Nocardia*, *Francisella tularensis* types A and B, and actinomycetes were used (5′ to 3′): GCATCGATGAAGAACGCAGC and TCCTCCGCTTATTGATATGC, 300 nM; ACCGACCACAAGGGG and GGTTGTAACCTCTTCGA, 300 nM; GAGACATCAATTAAAAGAAG and CCAAGAGTACTATTTCCGGTTGGT, 400 nM; CTTGTACTTTTATTTGGCTACTGAGAAACT and CTTGCTTGGTTTGTAAATATAGTGGAA, 300 nM; and GGATGAGCCCGCGGCCTA and CCAGCCCCACCTTCGAC, 400 nM, respectively. Each tube contained 10 *μ*L Real-time PCR Master Mix with EVAGREEN (GenMark, Turkey), 2 *μ*L primer mix, 3 *μ*L double-distilled water, and 5 *μ*L sample DNA. PCR was performed as described above. The specificities of the PCR products were assessed by melting curve analysis.

### 2.4. Statistical Analysis

All data were evaluated statistically. Correlations of the morphological characteristics with the histochemical and PCR data were analyzed. The chi-squared or Fisher's exact test was used to compare the frequencies of categorical variables, and Spearman's correlation test was used to determine the correlations between variables. A *p* value < 0.05 was considered statistically significant.

## 3. Results

**Figure 1:**
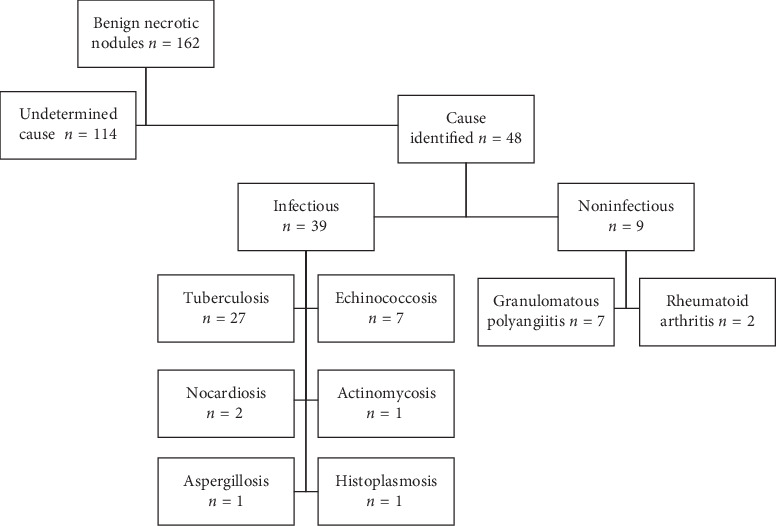
The study population.

We examined 162 patients with benign NNs. Of these, 48 were excluded because the NN etiology was determined based on clinical examination, radiological findings, microbiological or serological tests, or routine histopathological evaluation (Figure 1). The remaining 114 patients (60 males and 54 females) with NNs of unknown cause were analyzed. The mean (range) and median patient ages were 48.3 (20–79) and 50 years, respectively. The surgery performed was wedge resection in 97 (85%) and anatomical resection in 17 (15%).

**Figure 2:**
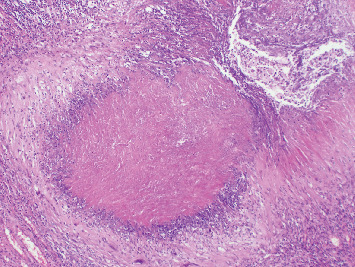
Nongranulomatous necrotic nodule, H&E, ×40.

**Figure 3:**
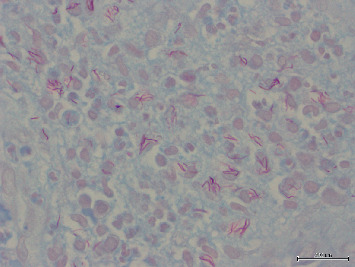
Acid-fast bacilli by EZN, ×1000.

NNs were classified as granulomatous in 90 patients (78.9%) and nongranulomatous in 24 (21%) (Figure 2). AFB were detected by EZN staining (Figure 3). PCR was used for MT and panfungal detection. The histochemical and PCR results are shown in Table 1.

Vasculitis was found in 29 cases. Lymphoplasmacytic vasculitis was detected in 89.6% (26). Granulomatous vasculitis was just in 10.3% (3) cases. There was no leukocytoclastic vasculitis and necrotic vasculitis.

Two patients exhibited foreign bodies and two thromboembolisms around the NNs. All four were negative for bacilli. Both patients with foreign bodies and one with a thromboembolism had granulomatous NNs.

MT was detected by PCR and/or EZN in 53.5% of all NNs: 62.2% of granulomatous NNs and 20.8% of nongranulomatous NNs. Both methods yielded positive results in 22 patients and negative results in 53 patients. Two patients were negative by PCR but positive by EZN staining, whereas 37 were positive by PCR but negative by EZN staining. We found a weak (Spearman's coefficient, *r* = 0.4) but highly significant (*p*=0.0001) correlation between the PCR and EZN staining results. Granulomatous NNs were significantly associated with other histopathological parameters and *Bacillus* positivity (Table 2).

We found a weak but significant correlation between granulomatous NN and *Bacillus* positivity, which was correlated significantly with the presence of a granuloma around the NN and the presence of multiple necrotic foci (*p* < 0.0001, *r* = 0.45; *p* < 0.0001, *r* = 0.28, respectively). *Bacillus* positivity was not correlated significantly with NN size, border type, shape, necrotic quality, vasculitis, or foreign body or thromboembolic status (*p*=0.49, *p*=0.57, *p*=0.61, *p*=0.33, and *p*=0.66, respectively).

Gram and Grocott staining did not identify a specific infectious cause in any case. Panfungal PCR was positive in two cases. PCR did not detect nontuberculous mycobacteria, *Nocardia*, *F. tularensis* type A or B, or actinomycetes in any case. Thus, the etiologies of benign pulmonary NNs were determined in 65 patients (57%) but not in 49 (42.9%) (Table 3).

## 4. Discussion

The leading causes of benign pulmonary NNs are infections (bacterial, fungal, and parasitic), vasculitis, pulmonary infarction, aspiration, rheumatoid arthritis, and necrotizing sarcoidosis [1–3]. NNs can be granulomatous or nongranulomatous. Necrosis commonly develops in patients with pulmonary granulomatous diseases and is caused by infection in nearly 50% of cases [4]. The coexistence of necrosis and a granuloma limits the number of etiological factors to consider during diagnosis. Indeed, diagnosis facilitates identification of the specific infectious agent [4]. Our results corroborate this, as the most common specific agent identified was MT. Pathological findings of granulomatous necrosis and a granuloma around the nodule are associated with a higher likelihood of tuberculosis. Lesions should be exhaustively tested for bacilli.

The diagnosis of pulmonary NN requires an algorithmic approach. Re-evaluation of new slides after histochemical staining, microbiological culture, fungal serological tests, PCR analysis, and clinical correlations all support a diagnosis. The specific agent can be determined retrospectively. Mukhopadya et al. reported that diagnosis was assisted by histological findings in 60% of cases, by microbiological culture in 47%, by fungal serology in 14%, and by clinical radiological confirmation in 8% [5]. PCR of NNs allows accurate detection of tuberculosis bacilli in the paraffin block [6]. Moreover, the importance of confirmation using other methods such as EZN staining has been emphasised [7–9]. Such staining is of undeniable diagnostic utility, being cost effective, rapid, and easy to perform [9]. Any step-by-step pathological evaluation should aim to detect tuberculosis bacilli.

In our study, vasculitis was detected in 25% of NNs. We observed no correlation between the granuloma status and *Bacillus* positivity. Thus, vasculitis is not specific in terms of NN differentiation. Perivascular and intramural inflammation may develop in patients with pulmonary tuberculosis and infarctoid necrosis [10]. In particular, the morphological lesions of granulomatous polyangiitis may not differ from lesions that develop secondary to infection [11]. Thus, the presence of vasculitis neither rules out nor compromises a preliminary diagnosis of tuberculosis.

Infectious granulomatous necrosis may mimic a thromboembolic infarction because the necrosis may be of a coagulative or dirty nature in the former condition [12]. Moreover, contrary to general belief, the borders of an infarct can be either irregular or round. Vascular inflammation may be evident. Activated epithelioid histiocytes at the border of an infarctoid necrosis render a definitive diagnosis difficult [13]. We found that granulomatous necroses containing bacilli can feature an infarctoid or even dirty necrosis.

Our study had certain limitations, of which the first is its retrospective design. Clinical data were obtained from files. However, in patients with conditions such as rheumatoid arthritis and vasculitis, serological tests may be negative during certain periods. Another limitation was that all patients underwent resection because malignancy was suspected; therefore, pre- or postoperative microbiological culture was not performed in all cases.

In conclusion, undetermined diagnoses of pulmonary NN need to be minimized. Causative agents may be detected via pathological evaluation of both granulomatous and nongranulomatous NNs. In particular, the presence of both a necrotizing granuloma and a granuloma in the adjacent parenchyma is particularly important. Even if bacilli cannot be detected, tuberculosis must be considered. Integration of pathological and microbiological methods is essential for diagnosis of pulmonary NNs.

## Figures and Tables

**Table 1:** Characteristics of the histopathological lesions.

Lesion	MT-positive/negative by PCR	AFB-positive/negative by EZN staining	Total MT-positive/negative
*Granulomatous NN* (*n* = 90)	54/36	21/69	56/34
*Nongranulomatous NN* (*n* = 24)	5/19	3/21	5/19
*NN number*			
1 (*n* = 53)	19/34	3/50	19/34
>1 (*n* = 61)	40/21	21/40	42/19
*NN size*			
≤1 cm (*n* = 35)	17/18	3/32	17/18
>1 cm (*n* = 79)	42/37	21/58	44/35
*NN border*			
Smooth (*n* = 52)	27/25	6/46	27/26
Irregular (*n* = 62)	32/30	18/44	34/28
*NN shape*			
Round (*n* = 57)	28/29	7/50	28/29
Triangular (*n* = 6)	3/3	1/5	3/3
Amorphous (*n* = 51)	28/23	16/35	30/21
*Necrotic quality*			
Caseified (*n* = 79)	46/33	22/57	48/31
Infarctoid (*n* = 17)	7/10	2/15	7/10
Nuclear debris (*n* = 18)	6/12	0/18	6/12
*Granuloma around the NN*			
Yes (*n* = 56)	42/14	19/37	44/12
No (*n* = 58)	17/41	5/53	17/41
*Vasculitis*			
Yes (*n* = 29)	12/17	8/21	14/15
No (*n* = 85)	47/38	16/69	47/38
*Surrounding parenchyma*			
Normal (*n* = 35)	19/16	6/29	19/16
Emphysema (*n* = 28)	12/16	3/25	12/16
Bronchiolitis (*n* = 15)	10/5	6/9	10/5
Lipoid pneumonia (*n* = 2)	1/1	0/2	1/1
Interstitial pneumonia (*n* = 30)	14/16	9/21	16/14
Interstitial fibrosis (*n* = 3)	3/0	0/3	3/0
Organized pneumonia (*n* = 1)	0/1	0/1	0/1

MT: *Mycobacterium tuberculosis*; EZN: Ehrlich–Ziehl–Neelsen stain; NN: necrotic nodule.

**Table 2:** Features of granulomatous necrotic nodules.

	Chi-squared *p* value	Correlation coefficient	Correlation *p* value
*Bacillus* positivity	**0.001**	**0.34**	**0.0001**
NN number	**0.001**	**0.31**	**0.001**
NN size	1	−0.01	0.94
NN border	0.41	0.14	0.15
NN shape	0.65	0.06	0.53
Necrotic quality	0.24	−0.13	0.18
Presence of granuloma around the NN	**0.0001**	**0.37**	**0.0001**
Vasculitis	0.40	0.10	0.27

NN: necrotic nodule.

**Table 3:** Pathological diagnosis of necrotic nodules.

	Granulomatous necrotic nodules	Nongranulomatous necrotic nodules
MT	56	5
MT + panfungal	1	—
Fungal	1	—
Infarct	1	1
Foreign body	2	—
Indeterminable	31	18

## Data Availability

The data used to support the findings of this study are available from the corresponding author upon request.
